# Cav-1 Ablation in Pancreatic Stellate Cells Promotes Pancreatic Cancer Growth through Nrf2-Induced shh Signaling

**DOI:** 10.1155/2020/1868764

**Published:** 2020-04-20

**Authors:** Shan Shao, Tao Qin, Weikun Qian, Xuqi Li, Wei Li, Liang Han, Dong Zhang, Zheng Wang, Qingyong Ma, Zheng Wu, Erxi Wu, Jianjun Lei

**Affiliations:** ^1^Department of Oncology, First Affiliated Hospital of Xi'an Jiaotong University, 277 West Yanta Road, Xi'an 710061 Shaanxi Province, China; ^2^Department of Hepatobiliary Surgery, First Affiliated Hospital of Xi'an Jiaotong University, 277 West Yanta Road, Xi'an, 710061 Shaanxi Province, China; ^3^Department of General Surgery, First Affiliated Hospital of Xi'an Jiaotong University, 277 West Yanta Road, Xi'an, 710061 Shaanxi Province, China; ^4^Department of Neurosurgery, Neuroscience Institute, Baylor Scott and White Health, Temple, Texas, USA

## Abstract

A more comprehensive understanding of the complexity of pancreatic cancer pathobiology, especially, and understanding of the role of the tumor microenvironment (TME) in disease progression should pave the way for therapies to improve patient response rates. Previous studies reported that caveolin-1 (Cav-1) has both tumor-promoting and tumor-suppressive functions. However, the function of Cav-1 in the pancreatic cancer microenvironment remains largely unexplored. Here, we show that coinjection of Cav-1-silenced pancreatic stellate cells (PSCs) with pancreatic cancer cells increased tumor growth. To comprehensively characterize paracrine communication between pancreatic cancer cells and PSCs, PSCs were cultured with pancreatic cancer cell conditioned medium (CM) containing cytokines. We reveal that Cav-1-silenced PSCs facilitated the growth of pancreatic cancer cells via enhanced paracrine shh/MMP2/bFGF/IL-6 signaling. Specifically, Cav-1-silenced PSCs exhibited increased shh expression, which heterotypically activated the shh signaling pathway in pancreatic cancer cells. Moreover, Cav-1-deficient PSCs accumulated ROS to enhance the shh pathway and angiogenesis in pancreatic cancer cells. In addition, overexpression of Nrf2 reversed the effects of Cav-1 knockdown on PSCs, increasing ROS production and enhancing paracrine shh/MMP2/bFGF/IL-6 signaling. Together, our findings show that stromal Cav-1 may mediate different mechanisms in the complex interaction between cancer cells and their microenvironment though Nrf2-induced shh signaling activation during pancreatic cancer progression.

## 1. Introduction

Pancreatic ductal adenocarcinoma (PDAC) is a recalcitrant malignancy with poor prognosis owing to inefficient diagnosis and strong drug resistance. Recently, the relationship between cancer cell progression and the tumor microenvironment (TME) has received increased attention [1–3]. Specifically, the cancer stroma, which is abundant in PDAC, was reported to contribute to tumor progression, invasion, metastasis, and chemoresistance [4, 5].

PDAC is characterized by the presence of an ample desmoplastic stroma, which constitutes up to 90% of the tumor volume [6, 7]. Cancer-associated fibroblasts (CAFs) are crucial for the formation of the desmoplastic stroma. In human pancreatic cancer, the main progenitors of CAFs are pancreatic stellate cells (PSCs) [8]. In the healthy pancreas, quiescent PSCs have a cytoplasmic lipid-storing capacity and are normally present in low numbers in the connective tissue, where they secrete only low levels of ECM components [9]. However, during malignancy, quiescent PSCs become activated, lose their cytoplasmic lipid-storing capacity, and secrete large amounts of ECM, which ultimately develops into a barrier for tumor drug penetration. In addition, activated PSCs generate a variety of cytokines and growth factors, which trigger tumor cells and other stromal cells, inducing tumor progression, metastasis, and drug resistance [10, 11]. Activated PSCs therefore present an attractive and promising cellular target for developing therapies to modulate the tumor stroma.

Caveolae are caveolin-1- (Cav-1-) enriched subdomains in the plasma membrane that are deregulated in cancer cells and contain a high cholesterol and sphingolipid content [12, 13]. Cav-1 is thought to be related to the regulation of many biological processes in both normal tissues and cancer [14, 15]. In general, Cav-1 has been reported to have both tumor-promoting and tumor-suppressive functions and is pro- or antisurvival depending on the cancer cell type [16–18]. Although previous studies have mainly focused on delineating the function of Cav-1 in cancer cells, recent studies have started to emphasize the function of the Cav-1 protein in the tumor microenvironment [19, 20]. In cancer-associated fibroblasts, Cav-1 underlies matrix stiffness and favors tumor invasion and metastasis [19], whereas others have shown that its loss in fibroblasts correlates with poor prognosis [21, 22]. Cav-1 is highly expressed in fibroblasts and endothelial cells, which are normally implicated in stromal remodeling during pancreatic cancer evolution [23]. In pancreatic cancer, knockdown of Cav-1 in fibroblasts led to enhanced tumor growth and chemoresistance [23]. The loss of stromal Cav-1 expression predicts poor clinical outcomes in pancreatic cancer [24].

To examine this issue, Cav-1 shRNA was applied to suppress PSC Cav-1 expression. In the experiments in which Aspc-1 cells were cultured with PSC conditioned medium (CM) or coinjected with PSCs, we showed that Cav-1-deficient PSCs accelerated the growth of pancreatic cancer cells *in vitro* and *in vivo* via paracrine shh signaling. In addition, Nrf2 was found to be responsible for the observed effects of the knockdown of Cav-1 in PSCs. Therefore, our data suggest that stromal loss of Cav-1 acts as a tumor promotor in pancreatic cancer progression.

## 2. Materials and Methods

### 2.1. Cell Culture and Reagents

The following antibodies were used: antibodies against Cav-1 (#3267), Nrf2 (#12721), cyclin A (#91500), Gli-1 (#2643), anti-mouse IgG HRP-linked secondary antibody (#7076), and anti-rabbit IgG HRP-linked secondary antibody (#7074) were obtained from Cell Signaling Technology (Danvers, MA, USA). Antibodies against shh (sc-373779), cyclin D1 (sc-8396), tubulin (sc-166729), and *β*-actin (sc-47778) were purchased from Santa Cruz Biotechnology (Dallas, TX, USA). Cyclopamine, an antagonist of SMO, was purchased from Selleck Chemicals (Houston, TX, USA). N-Acetyl cysteine (NAC) and DCF-DA were purchased from Sigma (Saint Louis, MO, USA).

### 2.2. Animal Studies

Pancreatic coinjection experiments were performed by subcutaneous coinjection of 5 × 10^6^ Aspc-1 cells with 0.5 × 10^6^ PSCs in the flanks of approximately 7-week-old BALB/c nude mice. The tumor volume in each animal was continuously monitored once a week for 5 weeks and calculated with the following formula: *V* (tumor volume) = 0.5 × *d* (short diameter)^2^ × *D* (long diameter). Then, all animals were killed. There were 6 mice in each group. All in vivo studies were approved by the Institutional Animal Ethical Committee of the First Affiliated Hospital of Xi'an Jiaotong University, China.

### 2.3. Cell Lines

Aspc-1 cells were purchased from the Cell Bank of the Chinese Academy of Sciences (Shanghai, China), and HUVECs were obtained from ALLCELLS (Emeryville, California, USA). In our experiments, only early-passage cells were used. Human PSCs were extracted from normal pancreatic tissues that had been isolated from donor patients who underwent liver transplantation at the Department of Hepatobiliary Surgery of the First Affiliated Hospital of Xi'an Jiaotong University. The isolation and PSC culture methods were as described in previous studies [8, 25, 26]. Morphological examination and oil red O staining of intracellular fat droplets were used to assess the purity of the PSCs, and *α*-smooth muscle actin expression in PSCs was assessed by immunofluorescence (see Supplementary Fig. S1). The PSCs used in this study were obtained from several patients. The written consent from patient's family was obtained, and the study protocol and consent forms were approved by the relevant ethical committee of the First Affiliated Hospital of Xi'an Jiaotong University, China.

### 2.4. Lentiviral Vectors

Stable knockdown of Cav-1 and GLi-1 was induced through lentiviral infection, and lentiviral particles containing negative control shRNA (sc-108080) (sh-Ctrl), shRNA (shCav-1) targeting Cav-1 mRNA (sc-270391-V), or shRNA against Gli-1 (sc-37912-V) were obtained from Santa Cruz Biotechnology (Dallas, Texas, USA). Cav-1- and Gli-1-interference efficiency in target cells was estimated by western blot analysis. In addition, Nrf2 overexpression vector-lentiviral activation particles (sc-400017) and negative control particles (sc-437282) were purchased from Santa Cruz Biotechnology. Lipofectamine was used to carry out transfection assay according to the manufacturer's instructions. After transfection, the stably transfected cells were selected by puromycin for further use.

### 2.5. Conditioned Medium Experiments

PSCs were maintained in 10% FBS-containing DMEM at 3 × 10^5^ cells per well in a 6-well plate for 12 h and then incubated in 1% FBS-containing DMEM for 24 hours. A total of 3 × 10^5^ Aspc-1 cells were incubated in the CM from these PSCs for 48 hours. Cyclopamine was obtained from Sigma and used to inhibit the shh pathway. Cyclopamine or dimethyl sulfoxide (DMSO) (Sigma) was added to the CM for 48 hours. Recombinant shh protein was obtained from PeproTech and used for hedgehog signaling activation in PSCs and Aspc-1 cells.

### 2.6. Enzyme-Linked Immunosorbent Assay (ELISA)

Cells (1 × 10^6^) were cultured in serum-free medium for 72 h, and then, the culture media were collected and centrifuged at 1500 rpm for 5 min to remove particles. The supernatants were collected and frozen at -80°C until use. The secretion of sonic hedgehog (shh), matrix metalloproteinase (MMP)2, basic fibroblast growth factor (bFGF), and interleukin 6 (IL-6) in the supernatants of PSCs was determined with a commercially available ELISA kit (R&D Systems, MN, USA) according to the manufacturer's recommendations.

### 2.7. Tumor Angiogenesis

A total of 5 × 10^6^ Aspc-1 cells and 0.5 × 10^6^ PSCs were implanted subcutaneously in the flanks of BALB/c nude mice. Three-micrometer-thick paraffin-embedded tumor sections were applied to assess the microvessel density (MVD) by CD31 (1 : 500; sc-1506) immunohistochemical staining [27]. The sections were imaged using an Axioplan Zeiss light microscope (Carl Zeiss, Ltd., Cambridge, UK) equipped with an AxioCam digital camera. CD31-positive vessels were calculated in 5 to 6 specimens per group (5 fields/sample). Whole-tumor lysates were collected to determine CD31 protein expression by immunoblotting [28, 29].

### 2.8. HUVEC Tubule Formation Assay

An in vitro angiogenesis assay was performed as previously described [30–32]. The wells in a 24-well plate were coated with 200 *μ*L of Matrigel each. A total of 2 × 10^4^ HUVECs per well were seeded into the coated plate with 200 *μ*L of the indicated CM described in the figure legends and cultured at 37°C under 5% CO_2_ for 24 h. Calcein-AM (0.2 *μ*M) was used to stain viable HUVECs at 37°C for 10 min. Capillary tube numbers were assessed by calculating the total tube length of each image at 100x magnification using a fluorescence microscope. Three different fields per well were randomly chosen and photographed. The total length of the capillary tubes within each field was calculated after calibration using a stage micrometer, and the analysis of the data was performed by GraphPad Prism 5 software (GraphPad Software, CA, USA).

### 2.9. [^3^H]Thymidine Incorporation Assay

The cells were plated (in triplicate for each experiment) in 24-well plates at a density of 2 × 10^4^ cells per well. DNA synthesis was determined by incubating asynchronously growing cells with 0.5 mCi/mL of [^3^H]thymidine (Perkin-Elmer) for 18 hours [33]. The growth of Aspc-1 cells was determined by MTT assay at 0, 24, 48, and 72 hours after seeding [21].

### 2.10. Western Blot

Tumor tissue samples and cells (1 × 10^6^) from each group were sonicated and lysed. Total protein was collected, and 100 *μ*g of each protein lysate was subjected to gel electrophoresis on 10% polyacrylamide-SDS gels under reducing conditions, transferred onto PVDF membranes, and then processed for immunoblot analysis as previously described [29]. Primary antibodies diluted at 1 : 1000 in 1× TBS-T were used overnight at 4°C under agitation. HRP-conjugated secondary antibodies diluted at 1 : 2000 in 1× TBS-T and supplemented with 5% nonfat milk (LabScientific, Highlands, NJ) were used at room temperature for 2 h prior to development using a Clarity Western ECL substrate (Bio-Rad). Then, the blots were developed using a Millipore ECL substrate. An enhanced chemiluminescence detection system was applied to image the immunoreactive bands.

### 2.11. Measurement of the Glutathione (GSH) Content

GSH and glutathione disulfide (GSSG) levels were measured in PSC (1 × 10^6^) extracts using the GSH reductase enzyme method [34, 35]. This assay is based on the reaction of GSH and thiols, which converts 5,5′-dithio-bis-(2-nitrobenzoic acid) (DTNB) to 5-thio-2-nitrobenzoic acid (TNB), which can be detected at *λ* = 412 nm. The test is specific to GSH due to the specificity of the GSH reductase enzyme to GSH: the rate of TNB accumulation is proportional to the concentration of GSH in the sample. Briefly, cell extract was diluted at 1 : 2 with KPE buffer (0.1 M potassium phosphate, 5 mM disodium EDTA, pH 7.5) prior to the addition of freshly prepared DTNB (2.5 mM) and GSH reductase (250 U/mL) solutions. Following the addition of *β*-NADPH, the absorbance (*λ* = 412 nm) was measured immediately at 30 s intervals for 2 min. The rate of change in absorbance was compared to that for GSH standards. The protocol used to measure GSSG in each sample was nearly identical to that used to measure GSH, except for pretreatment of the sample with 2-VP, which reacts out with GSH.

### 2.12. Invasion Assay

Invasion assays were conducted in 24-well Transwells with 8 *μ*m polyester membrane inserts (Millipore, Billerica, MA, USA). The upper surface of the membrane was coated with Matrigel (BD Biosciences, Franklin Lakes, NJ, USA). Aspc-1 cells from the indicated groups were plated at 1 × 10^5^ cells per Transwell insert with FBS-free DMEM in a 24-well plate, and 600 *μ*L of complete medium was added to the lower chamber. After incubation for 24 h, noninvaded cells on the top side of the filter membrane were removed by scraping with a cotton swab. Next, 4% paraformaldehyde was applied to fix the membrane, and crystal violet was used to stain the invaded cells. The invaded cells were counted and photographed under a light microscope. The average number of cells invaded per group was statistically analyzed.

### 2.13. Statistical Analysis

Data are represented as the means ± SEMs from at least three independent experiments. Statistical analysis was performed using GraphPad software. The specific analytical methods are detailed in the figure legends. Statistical significance was set at *P* < 0.05.

## 3. Results

### 3.1. Cav-1 Ablation in Pancreatic Cancer Stellate Cells Promoted the Growth of Pancreatic Cancer Cells *In Vivo*

**Figure 1:**
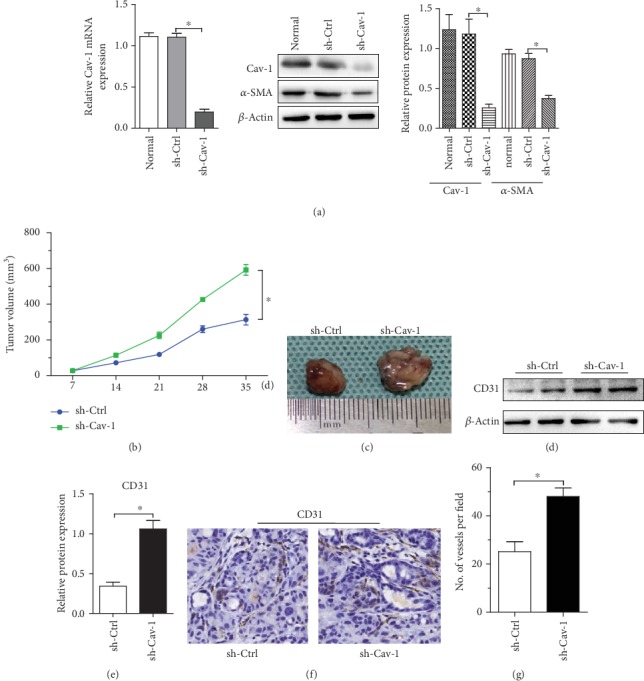
Cav-1 interference in PSCs facilitates Aspc-1 pancreatic cancer cell growth. (a) Cav-1 interference efficiency in PSCs was analyzed by qRT-PCR (left). Cav-1 and *α*-SMA protein expression in Cav-1-knockdown PSCs was analyzed by western blot (middle and right). sh-Ctrl stands for control shRNA. sh-Cav-1 stands for Cav-1 shRNA. (b) A total of 5 × 10^6^ Aspc-1 cells mixed with 0.5 × 10^6^ PSCs with or without Cav-1 knockdown (sh-Ctrl or sh-Cav-1) were implanted subcutaneously into the flanks of BALB/c nude mice (*n* = 6 per group). Tumor volumes were determined by measuring the width and length of the tumors every week. Mean (*n* = 6); bars, SD; representative images of tumors are displayed in (c). (d and e) CD31 immunoblot analysis of whole-tumor lysates is shown. (f and g) CD31 immunohistochemistry of tumor sections showing that microvascular density correlates with tumor size in the Cav-1-knockdown PSC group. The results shown are the means ± SEMs. ^∗^*P* < 0.05, by the two-tailed Mann–Whitney test and by Dunnett's multiple comparison test.

To determine whether the absence of Cav-1 in PSCs would affect Aspc-1 cell growth, Aspc-1+PSCs (with or without Cav-1 knockdown) were subcutaneously injected into the flanks of BALB/c nude mice. The Cav-1 interference efficiency and *α*-SMA protein expression were determined and are shown in Figure 1(a). After 5 weeks, the Aspc-1+Cav-1 knockdown PSC (sh-Cav-1) group exhibited an enhanced growth rate (~1.8-fold) compared with the Aspc-1 + normal PSC (sh-Ctrl) group (Figures 1(b) and 1(c)). Moreover, the MVD was increased in sh-Cav-1-group tumors compared to sh-Ctrl-group tumors, as determined using CD31 immunohistochemical staining analysis (Figures 1(f) and 1(g)). Simultaneously, western blot analysis showed that CD31 protein expression in sh-Cav-1-group tumors was significantly higher than that in sh-Ctrl-group tumors (Figures 1(d) and 1(e)). Taken together, these findings suggest that the accelerated growth of Aspc-1 cells by Cav-1-knockdown PSCs is correlated with their MVD.

### 3.2. Cav-1-Deficient Pancreatic Cancer Stellate Cells Displayed Increased Amounts of Protumorigenic Cytokines

**Figure 2:**
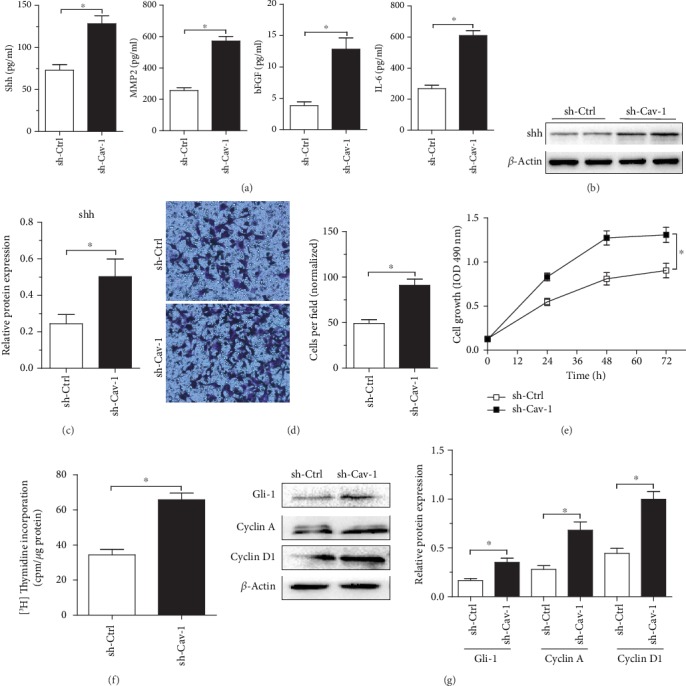
Cav-1-knockdown PSCs exhibit increased amounts of protumorigenic cytokines that promote pancreatic cancer growth and invasion. (a) shh, bFGF, MMP2, and IL-6 secretion in the CM of PSCs with or without Cav-1 knockdown (sh-Ctrl or sh-Cav-1) was determined by ELISA. (b and c) The results of shh immunoblot analysis of PSCs with or without Cav-1 knockdown (sh-Ctrl or sh-Cav-1) is displayed. (d) Aspc-1 cells were incubated with CM from PSCs with or without Cav-1 knockdown (sh-Ctrl or sh-Cav-1). The invasive ability of Aspc-1 cells was evaluated by counting the numbers of migrated cells in ten randomly selected fields under a light microscope at ×100 magnification. (e and f) MTT assay and [^3^H]thymidine incorporation assay (48 hours) in Aspc-1 pancreatic cancer cells treated with CM from PSCs with or without Cav-1 knockdown. (g) Immunoblot analysis showing increased expression of Gli-1, cyclin D1, and cyclin A in Aspc-1 pancreatic cancer cells incubated (48 hours) with CM from Cav-1-knockdown PSCs. Data represent the means ± SEMs. ^∗^*P* < 0.05, by two-tailed Student's *t*-test.

To evaluate whether Cav-1 expression mediates secreted soluble factors in PSCs, the levels of some cytokines in CM from normal PSCs (sh-Ctrl) and Cav-1-knockdown PSCs (sh-Cav-1) were determined. CM from sh-Cav-1 PSCs exhibited upregulated levels of shh, MMP2, bFGF, and IL-6, which exert proliferative, invasive, angiogenic, and inflammatory functions during pancreatic cancer progression (Figure 2(a)). As shown in Figures 2(b) and 2(c), the lysate of Cav-1-knockdown PSCs (sh-Cav-1) displayed higher shh protein expression than that of sh-Ctrl PSCs. Moreover, Aspc-1 cells cultured with CM from Cav-1-knockdown PSCs (sh-Cav-1) for 48 h exhibited enhanced cell invasion, growth/proliferation, and activation of shh signaling, as indicated by enhanced cyclin A/D1 and Gli-1 protein expression (a transcription gene in shh signaling) and by increased MTT and [^3^H]thymidine incorporation (Figures 2(d)–2(g)). Thus, our data suggest that Cav-1-deficient PSCs secrete cytokines that facilitate pancreatic cancer proliferation, invasion, and angiogenesis.

### 3.3. Suppression of shh Signaling in Pancreatic Cancer Cells Reversed the Proproliferative/Protumorigenic Effects of Cav-1-Deficient PSCs

**Figure 3:**
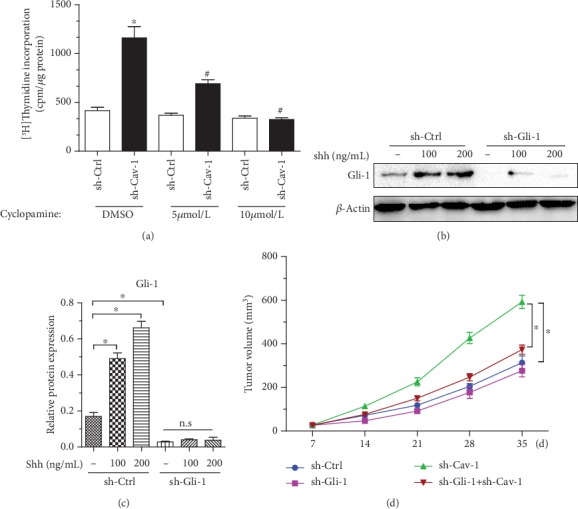
Suppression of shh signaling in pancreatic cancer cells abrogates the proproliferative/protumorigenic effects of Cav-1-knockdown PSCs. (a) [^3^H]Thymidine incorporation assay of Aspc-1 pancreatic cancer cells treated with DMSO or cyclopamine after incubation with CM from PSCs (sh-Ctrl or sh-Cav-1). ^∗^*P* < 0.05 versus the sh-Ctrl group; ^#^*P* < 0.05 versus the sh-Cav-1 group treated with DMSO. (b and c) Gli-1 and *β*-actin immunoblot analysis of sh-Ctrl- or sh-Gli-1-transfected Aspc-1 cells before and after treatment with shh. (d) A total of 5 × 10^6^ Aspc-1-sh-Gli-1 cells mixed with 0.5 × 10^6^ PSCs with or without Cav-1 knockdown (sh-Ctrl or sh-Cav-1) were implanted subcutaneously in the flanks of BALB/c nude mice (*n* = 6 per group). sh-Ctrl stands for coinjection of Aspc-1 cells and sh-Ctrl-transfected PSC group; sh-Gli-1 stands for coinjection of sh-Gli-1-transfected Aspc-1 cells and sh-Ctrl-transfected PSC group; sh-Cav-1 stands for coinjection of Aspc-1 cells and sh-Cav-1-transfected PSC group; sh-Gli-1+sh-Cav-1 stands for coinjection of sh-Gli-1-transfected Aspc-1 cells and sh-Cav-1-transfected PSC group; tumor volumes were determined by measuring the width and length of the tumors every week. Mean (*n* = 6); bars, SD; Gli-1 knockdown in Aspc-1 cells reversed the tumor-promoting effects of Cav-1-knockdown PSCs, as determined by coinjection experiments. The results are the means ± SEMs. *n* = 6 per group; ^∗^*P* < 0.05, by Tukey's multiple comparison test.

Aberrant activation of shh signaling has been shown in several cancer types, including pancreatic cancer [36]. To evaluate whether pharmacologic suppression of shh signaling in pancreatic cancer cells could reverse the effect of CM from Cav-1-knockdown PSCs on Aspc-1 proliferation, a [^3^H]thymidine incorporation assay was performed with pancreatic cancer cells cultured with CM from Cav-1-knockdown PSCs for 48 h with or without cyclopamine, a specific inhibitor of shh signaling. Our data revealed that 5–10 mM cyclopamine effectively inhibited the effects of CM from Cav-1-knockdown PSCs on Aspc-1 cell proliferation. Intriguingly, the proliferation of Aspc-1 cells cultured in CM from sh-Ctrl-transfected PSCs with cyclopamine remained unchanged (Figure 3(a)). To determine whether suppression of shh signaling in Aspc-1 pancreatic cancer cells abrogates the tumorigenic effects of Cav-1-knockdown PSCs *in vivo*, the Gli-1 gene was stably silenced through lentiviral shRNA transfection. Gli-1 expression was completely knocked down without exogenous shh stimulation, and decreased Gli-1 expression levels were achieved in the presence of exogenous shh (Figures 3(b) and 3(c)). Coinjection of PSCs and Aspc-1 cells showed that Gli-1 interference in Aspc-1 cells was sufficient to reverse the protumorigenic properties of Cav-1-knockdown PSCs (Figure 3(d)). These data indicate that shh signaling is pivotal for Cav-1-deficient PSC-induced Aspc-1 pancreatic cancer cell proliferation and tumor growth.

### 3.4. ROS Were Responsible for the Observed Effects of Cav-1-Deficient PSCs on Pancreatic Cancer shh Signaling and Angiogenesis

**Figure 4:**
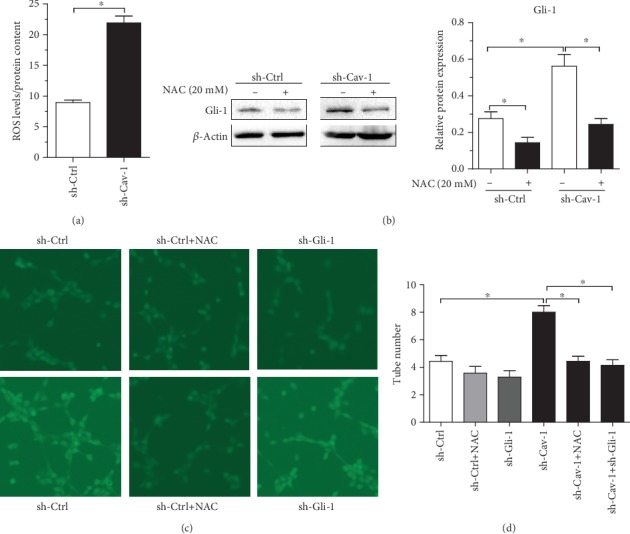
ROS are a key mediator in the observed effects of Cav-1-knockdown PSCs on shh signaling and angiogenesis in pancreatic cancer. (a) ROS production in PSCs with or without Cav-1 knockdown was evaluated with DCF-DA and normalized based on total protein content. (b) Gli-1 and *β*-actin immunoblot analysis of sh-Ctrl- or sh-Gli-1-transfected Aspc-1 cells cultured with CM from sh-Ctrl- or sh-Cav-1-transfected PSCs before and after treatment with NAC. (c and d) Angiogenesis was evaluated based on tube formation. HUVECs were cocultured with CM from the indicated groups. sh-Ctrl stands for CM from Aspc-1 cells cocultured with sh-Ctrl-transfected PSCs; sh-Ctrl+NAC stands for CM from Aspc-1 cells cocultured with sh-Ctrl-transfected PSCs and 20 mM NAC; sh-Gli-1 stands for CM from sh-Gli-1-transfected Aspc-1 cells cocultured with sh-Ctrl-transfected PSCs; sh-Gli-1+NAC stands for CM from sh-Gli-1-transfected Aspc-1 cells cocultured with sh-Ctrl-transfected PSCs and 20 mM NAC; sh-Cav-1 stands for CM from Aspc-1 cells cocultured with sh-Cav-1-transfected PSCs; sh-Cav-1+NAC stands for CM from Aspc-1 cells cocultured with sh-Cav-1-transfected PSCs and 20 mM NAC; sh-Cav-1+sh-Gli-1 stands for CM from sh-Gli-1-transfected Aspc-1 cells cocultured with sh-Cav-1-transfected PSCs; tube numbers were counted. Results are shown as the means ± SEMs; ^∗^*P* < 0.05, by two-tailed Student's *t*-test.

Since ROS are involved in the interaction between pancreatic cancer cells and PSCs [32], we examined whether ROS played a vital role in the observed effects of Cav-1-knockdown PSCs on pancreatic cancer shh signaling. N-Acetyl cysteine (NAC) was applied to reduce ROS in both PSCs and Aspc-1 cells. Cav-1-knockdown PSCs exhibited significantly increased ROS production compared with that in control (sh-Ctrl-transfected) PSCs (Figure 4(a)). Moreover, NAC abolished the ability of CM from Cav-1-knockdown PSCs to increase Gli-1 expression in Aspc-1 cells (Figure 4(b)). Furthermore, NAC reduced the proangiogenic effects of CM from Cav-1-knockdown PSCs on Aspc-1 cells (Figures 4(c) and 4(d)). However, NAC- and Cav-1-deficient PSCs could not suppress HUVEC tube formation when Gli-1 was knocked down in Aspc-1 cells (Figures 4(c) and 4(d)). These data suggest that ROS played a pivotal role in the observed effects of Cav-1-deficient PSCs on pancreatic cancer shh signaling and angiogenesis. Pancreatic cancer cell shh signaling mediates Cav-1-deficient PSC-induced angiogenesis.

### 3.5. Nrf2 Regulates Cav-1 Deficiency-Induced ROS Production and shh/MMP2/bFGF/IL-6 Secretion in PSCs

**Figure 5:**
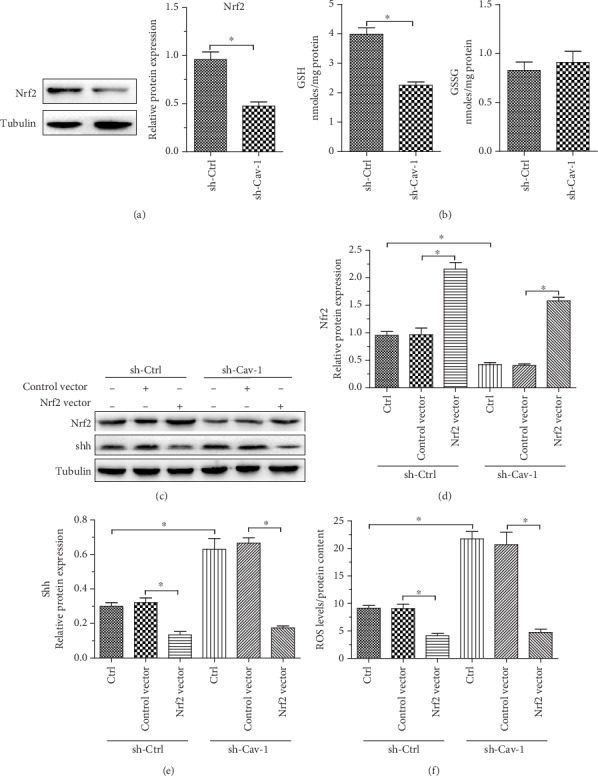
Nrf2 mediates ROS production induced by Cav-1 knockdown in PSCs. (a) Nrf2 protein levels in PSCs were determined by western blot. (b) GSH and GSSG levels were evaluated in PSCs (middle and right). ^∗^*P* < 0.05 versus the sh-Ctrl group (*n* = 3). All data are representative of at least three independent experiments. (c–e) Cav-1 in PSCs was silenced by transfection with control shRNA (sh-ctrl) or shRNA targeting Cav-1 (sh-Cav-1). Nrf2 overexpression DNA (Nrf2 vector) or control DNA (control vector) was added to the Cav-1-silenced group to determine the effect of Nrf2 overexpression on Cav-1-knockdown PSCs. Nrf2 and shh protein expression was detected by western blot. (f) ROS production in PSCs was evaluated with DCF-DA and normalized based on total protein content. Data represent the means ± SEMs. ∗ denotes *P* < 0.05, by two-tailed Student's *t*-test.

**Figure 6:**
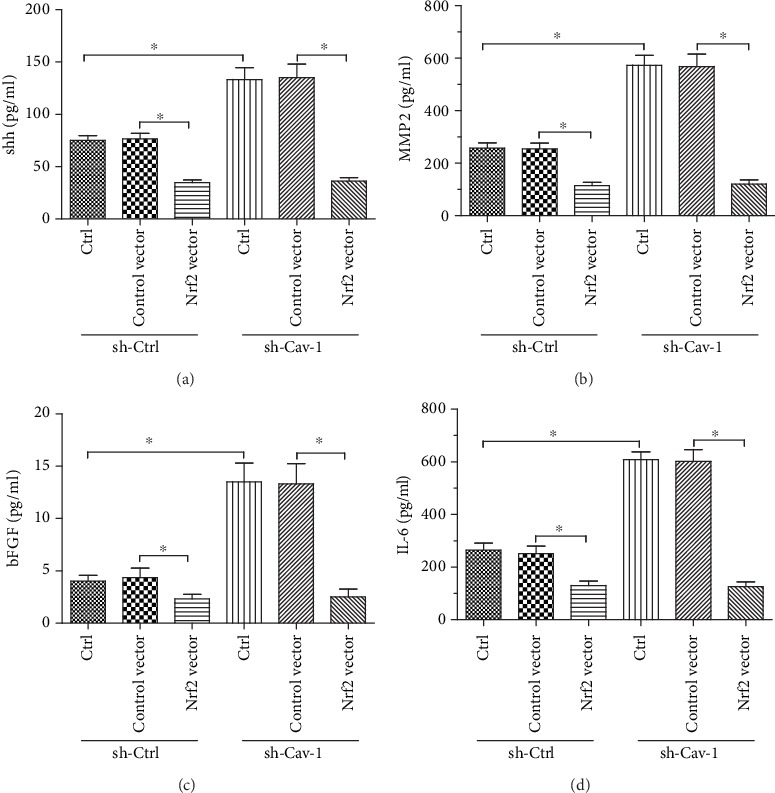
Nrf2 regulates shh/MMP-2/bFGF/IL-6 secretion elevated by Cav-1 knockdown in PSCs. Cav-1 in PSCs was silenced by transfection with control shRNA (sh-ctrl) or shRNA targeting Cav-1 (sh-Cav-1). Nrf2 overexpression DNA (Nrf2 vector) or control DNA (control vector) was added to the Cav-1-silenced group to determine the effect of Nrf2 overexpression on Cav-1 knockdown PSCs. The secretion of shh (a), MMP2 (b), bFGF (c), and IL-6 (d) was determined by ELISA. The results are shown as the means ± SEMs. ∗ denotes *P* < 0.05, by two-tailed Student's *t*-test.

Nrf2 plays a critical role in the cellular response to oxidative stress [37, 38]. We examined Nrf2 expression and GSH and GSSG expression in PSCs. Our data showed that Cav-1 interference induced a prominent reduction in Nrf2 and GSH expression without affecting GSSG expression (Figures 5(a) and 5(b)). Nrf2 overexpression suppressed the increase in shh protein expression induced by Cav-1 interference (Figures 5(c)–5(e)). Moreover, overexpression of Nrf2 reversed the effect of Cav-1 knockdown on PSC ROS production (Figure 5(f)). Intriguingly, the induced secretion of shh/MMP2/bFGF/IL-6 in Cav-1-deficient PSCs could be reversed by Nrf2 overexpression (Figure 6). These data indicate that Nrf2 is a critical regulator of Cav-1 knockdown-induced ROS production and shh/MMP2/bFGF/IL-6 secretion in PSCs.

## 4. Discussion

In our present study, we show that disruption of the Cav-1 gene accelerated the growth of Aspc-1 pancreatic cancer cells in mice and facilitated Aspc-1 cell invasion. Our data suggest that Cav-1 deficiency in PSCs is conducive to primary pancreatic cancer growth through upregulated paracrine cytokine signaling (including shh/MMP2/bFGF/IL-6) and that ROS play a vital role in the interaction between PSCs and pancreatic cancer cells. Moreover, Nrf2 is a critical regulator of Cav-1 knockdown-induced ROS production and shh/MMP2/bFGF/IL-6 secretion in PSCs.

Although the functions of Cav-1 in many tumor cells have recently been elucidated [39–41], the role of stromal Cav-1 in pancreatic cancer progression remains less well studied. Here, we reveal that tumor growth seen in coinjected Aspc-1 cells and Cav-1-silenced PSCs seems to be well associated with differences in their microvascular density. Our results are in agreement with previous studies demonstrating a direct positive correlation between the absence of Cav-1 and increased microvascular density in vivo [21, 42, 43]. PSCs are the main progenitors of CAFs. Activated PSCs generate a variety of cytokines and growth factors and exert important biological functions to maintain pancreatic cancer progression [44].

On this basis, we assumed that the lack of Cav-1 in PSCs would accelerate the growth of pancreatic cancer cells through paracrine signaling. To test this hypothesis, pancreatic cancer cells were cultured with CM from PSCs. Intriguingly, the CM culture assay indicated that the growth-promoting properties of PSCs lacking Cav-1 may be ascribed to increased paracrine signaling. As identified by ELISA, shh, MMP2, bFGF, and IL-6 secretion was significantly increased in Cav-1-deficient PSCs, which further confirms their protumorigenic phenotype. Additionally, these data are consistent with other studies that demonstrated the correlation of similar factors with microenvironment remodeling in tumors [21, 45]. A pivotal finding of this study is that the soluble form of the shh protein was upregulated in the CM of Cav-1-deficient PSCs. In addition to playing an essential role in embryonic development, shh signaling is closely involved in mediating pancreatic cancer growth, invasion, and metastasis [26, 46, 47]. Furthermore, there is growing evidence that shh may induce tumor growth in a paracrine manner [47, 48]. Our data revealed that CM from Cav-1-knockdown PSCs increased DNA synthesis and upregulated Gli-1 expression in pancreatic cancer cells. These results suggest that Cav-1 deficiency amplifies shh heterotypic signaling. As a consequence, the tumor growth-promoting feature of Cav-1-deficient PSCs is reversed through suppressing shh signaling with cyclopamine and by interfering with Gli-1 in Aspc-1 cells.

ROS are involved in pancreatic cancer EMT and invasion [26]. Here, we showed that Cav-1-deficient PSCs displayed significantly increased ROS production compared with that in normal PSCs. Inhibition of ROS production in Cav-1-knockdown PSCs suppressed increased Gli-1 expression in Aspc-1 cells. In addition, a similar effect on pancreatic cancer angiogenesis was observed. NAC and Cav-1-deficient PSCs could not suppress HUVEC tube formation when Gli-1 was silenced in Aspc-1 cells. These data suggest that Cav-1-deficient PSCs induce much more ROS to activate pancreatic cancer cell shh signaling in a paracrine manner, thus promoting cancer growth, invasion, and angiogenesis.

Previous studies showed that Nrf2 is a critical redox sensor and one of the master regulators of the antioxidant response [37, 49]. Nrf2 binds regulatory antioxidant response elements and activates the transcription of many antioxidant genes that counteract ROS [50]. In this study, we demonstrate that Cav-1 deficiency in PSCs markedly induced a prominent reduction in Nrf2 and GSH expression without affecting GSSG expression. These results indicate that silencing Cav-1 enhances PSC oxidative stress by downregulating Nrf2 expression. Overexpression of Nrf2 reversed the effect of Cav-1 knockdown on PSC ROS production and shh/MMP2/bFGF/IL-6 secretion. These findings indicate that Nrf2 plays an essential role in the effects of Cav-1-knockdown PSCs on pancreatic cancer progression.

In conclusion, we reveal that ablation of Cav-1 in PSCs promoted the growth, invasion, and angiogenesis of pancreatic cancer. Mechanistically, this feature may be attributed to enhanced ROS production and paracrine cytokine signaling (shh/MMP2/bFGF/IL-6) in Cav-1-deficient PSCs. Nrf2 is a key mediator in Cav-1 knockdown-induced ROS production and shh/MMP2/bFGF/IL-6 secretion in PSCs. Therefore, our findings add new evidence that importance must be attached to the complex interactions between cancer cells and their microenvironment for effective anticancer therapy development.

## Data Availability

All data generated during this study are included in this article.

## Supplementary Materials

Supplementary Materials Figure S1 A PSCs were isolated from normal pancreata removed from patients who underwent liver transplantation. Oil red O staining was performed to identify PSCs. B, C, and D *α*-SMA expression in PSCs was determined by immunofluorescence microscopy analysis.
