# Supplementary material for: Cav-1 Ablation in Pancreatic Stellate Cells Promotes Pancreatic Cancer Growth through Nrf2-Induced shh Signaling
Source: Oxid Med Cell Longev. 2020 Apr 20;2020:1868764. doi: 10.1155/2020/1868764 (PMC7189317; doi:10.1155/2020/1868764)
Supplement: Supplementary Materials — Figure S1 A PSCs were isolated from normal pancreata removed from patients who underwent liver transplantation. Oil red O staining was performed to identify PSCs. B, C, and D α-SMA expression in PSCs was determined by immunofluorescence microscopy analysis. [file 1868764.f1.doc]

Supplementary materials

Figure S1


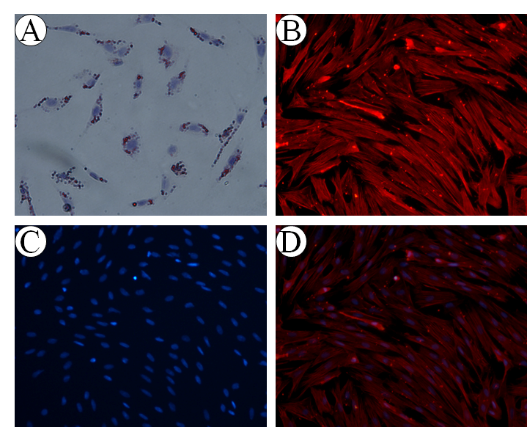


Figure S1.

A. PSCs were isolated from normal pancreata removed from patients who underwent liver transplantation. Oil red O staining was performed to identify PSCs. B, C & D. α-SMA expression in PSCs was determined by immunofluorescence microscopy analysis.
